# Schwannomas in the head and neck: retrospective analysis of 21 patients and review of the literature

**DOI:** 10.1590/S1516-31802007000400005

**Published:** 2007-07-01

**Authors:** Erwin Langner, André Del Negro, Hugo Kenzo Akashi, Priscila Pereira Costa Araújo, Alfio José Tincani, Antonio Santos Martins

**Keywords:** Neurilemmoma, Peripheral nerves, Myelin sheath, Neurofibromatoses, Head and neck neoplasms, Neurilemoma, Nervos periféricos, Bainha de mielina, Neurofibromatose, Neoplasias de cabeça e pescoço

## Abstract

**CONTEXT AND OBJECTIVE:**

Schwannomas are benign neoplasms of the peripheral nerves originating in the Schwann cells. According to their cellularity, they can be subdivided into Antoni A or Antoni B types. They are rare and usually solitary, with clearly delimited capsules. They occur in the head and neck region in only 25% of the cases, and may be associated with Von Recklinghausen's disease. The present study retrospectively analyzed some data on this disease in the head and neck region and reviewed the literature on the subject.

**DESIGN AND SETTING:**

Retrospective study at Head and Neck Service, Universidade Estadual de Campinas.

**METHODS:**

Data on 21 patients between 1980 and 2003 were reviewed. The sites of cervical schwannomas and the intraoperative, histopathological and postoperative clinical status of these cases were studied. Diagnostic methods, type of surgery and association with neurofibromatosis were evaluated.

**RESULTS:**

The patients’ ages ranged from 16 to 72 years. Four patients had a positive past history of type I neurofibromatosis or Von Recklinghausen's disease. The nerves affected included the brachial and cervical plexuses, vagus nerve, sympathetic chain and lingual or recurrent laryngeal nerve. The nerve of origin was not identified in six cases. Tumor enucleation was performed in 16 patients; the other five required more extensive surgery.

**CONCLUSION:**

Schwannomas and neurofibromas both derive from Schwann cells, but are different entities. They are solitary lesions, except in Von Recklinghausen's disease. They are generally benign, and rarely recur. The recommended surgical treatment is tumor enucleation.

## RESUMO

**CONTEXTO E OBJETIVO:**

Schwannomas são neoplasias benignas dos nervos periféricos, originadas nas células de Schwann, podendo, baseado na sua celularidade, ser subdivididas em Antoni A ou B. São de ocorrência infreqüente, geralmente únicas, com cápsula bem delimitada; ocorrendo na região de cabeça em pescoço em apenas de 25% dos casos, podendo estar relacionadas à doença de Von Recklinghausen. Este estudo objetiva analisar retrospectivamente dados concernentes a esta afecção na região de cabeça e pescoço e rever os dados da literatura.

**TIPO DE ESTUDO E LOCAL:**

Estudo retrospectivo realizado no Serviço de Cirurgia de Cabeça e Pescoço, Universidade Estadual de Campinas.

**MÉTODOS:**

Revisamos dados de 21 pacientes tratados entre 1980 e 2003. Sítio do tumor, status intra e pós-operatório e laudo histopatológico foram estudados nos schwannomas cervicais. Métodos diagnósticos, tipo de cirurgia e associação com neurofibromatose foram avaliados.

**RESULTADOS:**

A idade variou entre 16 e 72 anos. Quatro pacientes tinham história positiva para neurofibromatose tipo I ou doença de Von Recklinghausen. Os nervos afetados incluíram os plexos braquial e cervical, os nervos vago, lingual e recorrente e a cadeia simpática. O nervo de origem não foi identificado em seis casos. Enucleação do tumor foi feita em 16 pacientes; os outros cinco requereram cirurgias mais extensas.

**CONCLUSÃO:**

Schwannomas e neurofibromas derivam das células de Schwann, mas são tumores diferentes. São lesões solitárias, exceto na doença de Von Recklinghausen, geralmente benignas e raramente recidivam. O tratamento cirúrgico recomendado é a enucleção do tumor.

## INTRODUCTION

Among the benign tumors of the peripheral nerves, one specific group originates from Schwann cells. The group is currently divided into two subtypes: neurofibromas and schwannomas. Schwannomas are characterized by the presence of specific hypercellular areas known as Antoni A areas, with frequent nuclear palisading arrangements (Verocay bodies), and by less dense reticular areas called Antoni B or reticular-type areas.^1,2^ They are rare neoplasias, and are usually single encapsulated lesions. They occur in the head and neck region in approximately 25% of the cases, and are sometimes associated with Von Recklinghausen's disease.^3,4^

## OBJECTIVE

The present study aimed to retrospectively describe our experience with 21 patients with head and neck schwannomas, the diagnostic methods used, the surgical decisions and the treatment outcome, and to analyze the data and review the literature available on this type of tumor.

## MATERIAL AND METHODS

Between 1980 and 2003, 30 consecutive cases of benign masses in the head and neck that were suspected to be benign schwannomas were treated at the Head and Neck Surgery Service of the Department of Surgery, Universidade Estadual de Campinas (Unicamp), Brazil.

The inclusion criteria for this study were that the patients should not have had any previous treatment and that their postoperative histological diagnosis confirmed that the mass was a schwannoma. In the end, nine patients were excluded: five had had previous surgical treatment and three did not have histological confirmation that the mass was a schwannoma (in these cases, the diagnosis was plexiform neurofibroma), while one patient refused surgical treatment.

The preoperative diagnostic tests always included imaging examinations, and computed tomography was the method most commonly used (nineteen cases). The other imaging examinations used were magnetic resonance (MRI) in four cases and arteriography in two cases. Fine-needle aspiration biopsy was used in twelve cases.

The treatment of choice was conservative surgery, in which the main nerve trunk was preserved whenever feasible, with resection of the mass alone. Postoperatively, the data on surgical complications and nerve deficits caused by tumor resection were evaluated.

A review of the literature available on the topic was also carried out.

## RESULTS

Among the 21 patients analyzed, 13 (61.9%) were men. The patients’ ages ranged from 16 to 72 years. Three patients (14.2%) were aged 20 years or under, ten (47.6%) were between 21 and 40, five (23.8%) between 41 and 60, and three were over 60. Four patients (19%) had family or personal histories of type I neurofibromatosis (Von Recklinghausen's disease), but there was no association with other preexisting conditions.

For propaedeutic purposes, fine-needle aspiration biopsy was performed in 12 cases. In nine of these cases (75%) the results were inconclusive, and in the remaining three (25%) the results differed from the findings obtained from the surgical specimen.

**Figure 1.**
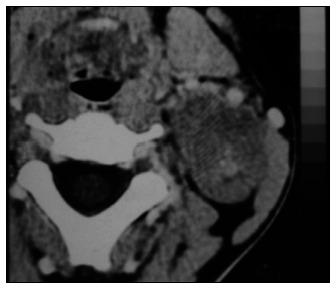
Cervical computed tomography scan showing schwannoma in sympathetic chain.

**Figure 2.**
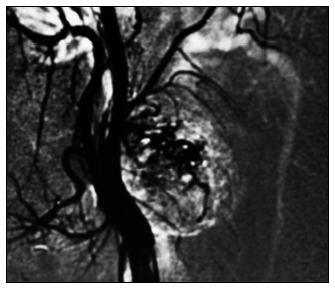
Arteriogram showing the same lesion as in Figure 1.

Among the nerves affected, the brachial plexus was the tumor site in five cases (23.8%), the vagus nerve in three (14.2%) and the sympathetic chain in four (19%) (Figures 1 and 2). There was also one case affecting each of the lingual, recurrent laryngeal and cervical plexus nerves, respectively (4.7% each). It was not possible to identify the nerve of origin in six cases (28.6%) (Table 1).

**Table 1.** Distribution of schwannomas by anatomical site among

Site	n (%)
Brachial plexus	5 (23.8%)
Vagus nerve	3 (14.2%)
Sympathetic chain	4 (19%)
Unidentified	6 (28.4%)
Others	3 (14.2%)

The surgical procedure performed in 16 cases consisted of enucleation of the neoplasia. In three patients (14.2%), associated (cervical or axillary) node dissection was performed. In five cases gross intraoperative perineural invasion was noted, requiring complete resection of the neural segment involved.

Nine patients (42.8%) presented neurological sequelae during the postoperative evolution. Of these, four underwent complete resection of the nerve and five underwent tumor enucleation. Five of the nine cases of neurological sequelae (55.5%) were associated with Horner's syndrome (due to resection of the sympathetic chain). Two patients (9.5%) developed postoperative dysphonia. In three cases (14.2%), the anatomopathological examination indicated the presence of malignant schwannoma.

During the clinical follow-up three patients died: one due to hemorrhage during surgery, another from postoperative pneumonia and the third due to local-regional recurrence (malignant schwannoma). The average length of follow-up for the 19 surviving patients was 6.2 years. During this period, clinical examinations showed that five patients (23.8%) developed new neurogenic tumors.

## DISCUSSION

According to the literature,^1-4^ schwannomas are equally distributed between the genders, and this finding was confirmed by the present study. In terms of age, the greatest incidence in our study was between the third and fifth decades, which has also been described by other authors.^4-8^

The incidence of type I neurofibromatosis in such cases has ranged from 8% to 18% in the literature,^3,4,8,9^ and was found in 19% of the cases studied here. In cases unassociated with neurofibromatosis, schwannomas are seen clinically as solitary slow-developing lesions that show symptoms only when large areas have been affected.

Fine-needle aspiration biopsy has drawbacks in terms of accuracy, as described by Zbären et al.,^7^ and does not constitute an effective means of preoperative diagnosis.

The low incidence of schwannomas is also reflected when the anatomical sites involved are considered. In the case of the brachial plexus (five cases in our study), 147 cases had been published up to 1987.^8^ The cranial pairs most affected by neurilemmomas are the ninth, seventh, eleventh, fifth and fourth, in order of frequency.^10^ The vagus nerve, which was involved in 14.2% of our study, can be said to be an infrequently affected site: up to 1989 approximately 70 cases had been described in the literature;^3^ the first was described by Sekiguichi in 1926. Schwannomas of the sympathetic chain are even rarer, responsible for four cases in our study, and only 14 cases had been described up to 1997.^5^ Other infrequent sites include the lingual and recurrent laryngeal nerves, cervical plexus, nasal and paranasal regions,^11^ facial nerve,^11^ posterior pharyngeal wall,^12^ larynx,^2^ thyroid gland^13^ and other regions. According to Seppälä, multiple lesions may occur in 3% to 4% of the patients with schwannomas.^8^ It is sometimes difficult to obtain a differential diagnosis between the originating sites.^14^

The classical surgical treatment is enucleation of the tumor, with care always taken to preserve the function of the affected nerve. According to Valentino,^15^ complete excision of the affected nerve was performed in 56% of the cases reported in the literature. Among the patients in whom the nerve was preserved, 64% developed permanent deficits and 29% transitory deficits.

According to the literature, approximately 4% of malignant schwannomas occur in cervical nerves.^15^ In our study, the percentage of malignant lesions was 14.2%, possibly because the cases occurred at a regional referral hospital. Malignant schwannoma tumors differ from the benign type in their higher mitotic rate, the presence of necrosis, their infiltrative appearance and irregular positivity for the S-100 protein.^16^ Surgery is almost always the treatment of choice, since radiotherapy and chemotherapy are of limited effectiveness in these cases.

## CONCLUSION

Schwannomas and neurofibromas are both derived from Schwann cells, but they differ from each another. They are usually solitary lesions, except in Von Recklinghausen's disease. Pain and neurological symptoms are uncommon, and become evident only at advanced stages. They are generally benign, and rarely recur. Preoperative diagnosis is sometimes difficult and differential diagnoses are widely variable. The possibility of neurological sequelae should always be explained during the preoperative interview with the patient. Surgical treatment does not always fully eliminate the neoplasia. On the other hand, because of its slow growth, it is unusual to observe local/regional recurrence. There has been very little discussion about these tumors in the literature, and their distribution is also controversial because of the small number of cases described.
